# Proper Clothing

**Published:** 1890-09

**Authors:** 


### PROPER CLOTHING.

No one will deny the position, second to none, which dress occupies in the administration of necessity, comfort and convenience to mankind, and yet one does constantly overlook it, by ignoring all its claims except as they are imposed by fashion. The idea of the real purpose of clothing enters little into the purchasing of material for, or the making of, a new gown. If it meet the demand of the times in fabric, texture, and color, it is taken regardless whether or not its heat conductive or non-conductive qualities are suited to the season or to the person's relative cold or warm "bloodedness"; and in the style advised by the *modiste* to set off a good figure or conceal a poor one, it is made, regardless of the health, comfort, or convenience of the wearer.

The absurdities of this custom will send one for an afternoon's promenade on a hot summer's day, in an *au fait* broadcloth street suit, heavily garnished, and in the cool of the evening to a lawn party, in the thinnest of thin mull draperies, half denuded of the underclothing in the bargain, the better to show off the round whiteness of arms and throat, more than suggested through veilings of filmy lace. The appropriateness of the garment to the condition of the weather or the wearer seems never to be taken into consideration ; if it conform to the latest whim for the occasion, it is enough.

Now it does not signify that because a dress is suitable for one season, it is equally suitable for another ; or even that a garment perfectly comfortable for one stage of a day's wear may meet the requirements of another portion of the same day. Changes in the clothing should be made in conformity to changes in the temperature, even in the course of a few hours ; for it is no rare thing in our climate for a day to run the gauntlet of forty degrees' difference. Neither can thin sensitive persons emulate with safety those through whose veins the rich blood warmly courses, in appropriating the coolest of materials, even in the height of the heated season.

Persons who have a care for health and comfort, then (and such are generally those who have a care for the exhibition of propriety and good sense, also), when they purchase material, will do so with a view to the uses to which it is to be put ; and reference to these uses will be carried out in the making. Other considerations beside mere appearance will enter into the wearing of garments, and they will be changed as many times a day as the wearer's comfort and the state of the weather may necessitate.—From *Good Health.*

